# The Medico-Psychological Association

**Published:** 1910-07-30

**Authors:** 


### THE MEDICO-PSYCHOLOGICAL ASSOCIATION.

The sixty-ninth annual meeting of the Association was held at the Royal College of Physicians and Morningside Asylum, Edinburgh, on Thursday and Friday, July 21 and 22. The first sitting was occupied with the business and administrative affairs of the Association, the only item of public interest being a modification of the entrance conditions for the nurses' examination. It was agreed that the candidates should not be less than twenty-one years of age at the final examination, and that nurses who entered the asylum service prior to November 1, 1909, should be eligible to enter for the nursing examination under the old regulations if they present themselves before November 1912. The first function after luncheon was the retirement from the Presidency of Professor Bevan-Lewis, M.Sc., and the induction of his successor, Dr. John Macpherson, of Edinburgh. The new President delivered his inaugural address, and a large number of papers were read during the meetings, the majority on bacteriological and bio-chemical subjects, which cannot be done justice to in a short *résumé*.

### Lunacy Administration in Scotland.

A contribution of much general interest was one by that veteran alienist, Dr. A. R. Urquhart, of the Murray House Asylum, Perth. His subject was "Lunacy Administration in Scotland, with special reference to the Royal Asylums." The first part of the paper consisted of an interesting historical survey of the methods of treating insane patients. Scotland was fortunate in its legal system, which was elastic enough to permit of the adoption of new ideas as they matured. The legal representative in each county or group of counties was the sheriff, who dealt with such cases as might be submitted to him. In that way legal matters were dealt with by lawyers who were competent and accessible. Their courts commanded the respect and confidence of the people. An Act in 1815 put lunacy administration into some order, for sheriffs were empowered to grant licences for keeping asylums, and inspectors were elected. Sheriffs were allowed to grant orders for detention of insane persons on medical certificates, and to ascertain if they were properly confined. But the results were not satisfactory, as many of the insane were in a deplorable condition until the middle of the century. To Miss Dix, "the American Invader," in 1855, belonged the credit of obtaining a Royal Commission of Inquiry, on the report of which Commission the present Lunacy Law of Scotland was established.

### Proportional Statistics.

Dr. Urquhart presented the following statistics, showing the distribution of the insane on January 1, 1858, and January 1, 1909 :—

| Class | Year | Public Asylums | Private Asylums | District Asylums | Idiot Schools | Poorhouses and Parochial Asylums | In Private Houses | Total |
|---|---|---|---|---|---|---|---|---|
| Private | 1858 | 786 | 196 | 0 | 23 | 6 | ? | 1,011 |
| | 1909 | 1,940 | 90 | 329 | 204 | 0 | 119 | 2,682 |
| Pauper | 1858 | 1,594 | 520 | 0 | 6 | 833 | 1,784 | 4,737 |
| | 1909 | 1,701 | 0 | 9,380 | 261 | 1,296 | 2,877 | 15,515 |

or

| Year | Males | Females | Total | Proportion to Population |
|---|---|---|---|---|
| 1858 | 2,718 | 3,030 | 5,748 | = 1 in 522 |
| 1909 | 8,892 | 9,305 | 18,197 | = 1 in 268 |

There was thus a great increase in reported insane persons between 1858 and 1909; and an increase in the private patients. There had been a development of district asylums with a certain proportion of private patients. There had also been a great increase of idiots in training schools, as well as in licensed poorhouses, and in the number of boarded-out pauper insane. There were signs that the high-water mark for Scotland had been reached. The Commissioners gave the following table of the proportion of insane per 100,000 of the estimated population in Scotland since 1904 :—

| January 1 | Private | Pauper | Total |
|---|---|---|---|
| 1904 | 52 | 307 | 359 |
| 1905 | 52 | 311 | 363 |
| 1906 | 51 | 312 | 363 |
| 1907 | 50 | 312 | 362 |
| 1908 | 51 | 314 | 365 |
| 1909 | 51 | 315 | 366 |

The Lunacy Act provided for urgently required accommodation by the erection of district asylums. The majority of the private asylums of 1855 were unsuitable, and many of them were abolished, and with that step a dark chapter in Scottish history was closed, the shameless competition for unfortunate persons at rates of maintenance which could not be regarded as sufficient was terminated by steady calculated pressure which extinguished the unfit. The Scotch parochial asylums were few. They were practically poorhouses with unrestricted licences. At present there was only one parochial asylum left.

### The Boarding-Out System.

Dr. Urquhart proceeded to discuss the boarding-out system.

With regard to the poor private insane, the following figures showed the rates of board per annum charged by the Royal Asylums so as to meet the patients' narrow means. Aberdeen, £30; Dumfries, £25; Dundee, £25; Edinburgh, £32 10s.; Glasgow, £26; Montrose, £25; Perth, £30. In 1855 it was found that the total capital expenditure made by the Royal Asylums for lands, buildings, and furniture amounted to £352,632, which sum had steadily increased, largely by the surplus profits derived from payments by well-to-do patients, until it now amounted to more than a million pounds.

### The Treatment of Mental Excitement.

Another paper of great interest was that by the medical superintendent of Craig House Asylum, Morningside. Dr. George Robertson, in introducing a discussion on the treatment of mental excitement in asylums, said that of cases of mental excitement in asylums it might be said "they are always with us," and the manner in which they were treated and the success attending their treatment might be taken as tests of the good management of an asylum. The difference between the state of the mad-houses of the past and of the mental hospitals of the present day was largely the result of better methods of dealing with excitement. Subjecting mental excitement to analysis, Dr. Robertson divided it into two kinds. There was, on one hand, the mental excitement which was directly due to disease, as of the person who suffered from acute mania. There was, on the other hand, the mental excitement which was a reaction to some irritation in the environment acting upon excitable patients. The obvious treatment for this secondary excitement in excitable patients was preventive. Referring briefly to the treatment of mental excitement, which was a direct symptom of such diseases as acute mania, agitated melancholia, and delirious insanity, he said that in these diseases sedative drugs were often employed. As their use was condemned by some, he mentioned the advantages and disadvantages of their employment. With certain precautions, he had not hesitated to employ sedatives, but there were some who totally abstained from doing so. All measures directed to improvement of the general health of the patient acted indirectly on excitement by tending to shorten the course of the disease.

Dr. Robertson discussed in detail the measures for prevention of secondary excitement; or, in other words, the management of irritability and excitability, which was a much more important problem than the treatment of the essential excitement of disease. In every asylum this preventable excitement was in excess of what it should be, and in the past it was very largely so. One of the worst manifestations of excitement was noisiness, and it was not sufficiently realised and acted upon that noise in an asylum was as infectious as measles in a preparatory school. It was, therefore, necessary to start a crusade against noise if one desired to abolish excitement in an asylum. Even shouting out requests or directions by the staff should not be permitted, and he had to record that he came to regret the day when he had furnished every ward in the Stirling District Asylum with a piano, on account of the exciting influence of music as usually played in an asylum. In cases of excitement, where the patient was confused, demented, or imbecile, the task of finding out the exciting cause was more difficult, but it should, nevertheless, be regarded as a duty, for even in these cases, although apparently hopeless, much of the excitement was preventable. Violent assaults on the part of irritable male patients, resulting in the use of an unnecessary degree of controlling force by male attendants, were features of excitement on the male side which gave rise to most anxiety in asylum administration. It was not too much to say that nine-tenths of the really serious incidents (excluding suicides) which the authorities had to investigate were of this nature. With the general improvement in efficiency which had taken place by the training of the staff, by more reliable supervision, and by higher ideals of care and treatment, violence of conduct had diminished very greatly in recent years. To his certain knowledge, no single factor exerted a more powerful influence in restraining this violence and intemperate language than the presence and influence of female nurses in the male wards. That this should be so was only what one would naturally expect, and this was the reason why during seventy years innumerable, but only partially successful attempts had been made to employ women in this manner. This system had now been successfully inaugurated by the superintendents of the Scottish asylums, with the warm approval and encouragement of the Commissioners in Lunacy. In conclusion, he emphasised that the greater proportion of the excitement found in asylums was of a preventable kind, and that this excitement could be prevented by minutely analysing the mental state of excitable patients, and by adopting treatment suitable for each case.

